# Brave new epigenomes: the dawn of epigenetic engineering

**DOI:** 10.1186/s13073-015-0185-8

**Published:** 2015-08-04

**Authors:** Anna Köferle, Stefan H. Stricker, Stephan Beck

**Affiliations:** Medical Genomics, UCL Cancer Institute, University College London, 72 Huntley Street, London, WC1E 6BT UK; Functional Epigenetics, Institute of Stem Cell Research, Helmholtz Zentrum, German Research Center for Environmental Health, Ingolstädter Landstrasse 1, 85764 Neuherberg, Germany; Biomedical Center, Ludwig-Maximilian-Universität, Grosshaderner Strasse 9, 82152 Planegg-Martinsried, Germany

## Abstract

New methods for epigenome editing now make it possible to manipulate the epigenome in living cells with unprecedented specificity and efficiency. These ground-breaking approaches are beginning to yield novel insights into the function of individual chromatin marks in the context of cellular phenotype.

## Enabling technologies

Our desire to manipulate genomes is not new; selective breeding, genetic and, more recently, genome engineering have greatly advanced our understanding of how genes shape phenotypes. On the cellular level, however, differences in phenotype are mainly determined by epigenetic processes. Millions of chromatin marks have been profiled across the human genome in different tissues and cell types. Yet, we are just beginning to understand how chromatin-based mechanisms contribute to the establishment, control and inheritance of gene activities. Epigenetic research has long been hindered by the lack of experimental methods that would allow the targeted manipulation of chromatin marks in living cells, since mutational approaches and pharmacological inhibition alter marks globally and nonspecifically. Epigenome engineering now fills this gap, making it experimentally possible to modify individual chromatin marks at specific user-defined sites. Targeted modification is achieved by fusing an effector, usually an endogenous chromatin-modifying enzyme or its minimal catalytic domain, to a programmable DNA-binding domain. The DNA-binding domain may be a synthetic zinc finger (ZF) or a TAL protein (TALE), which can be engineered to bind to a DNA sequence of choice. Alternatively, the chromatin modifier is fused to a catalytically inactive version of the Cas9 protein (dCas9), which is targeted via a separate, synthetic RNA molecule known as the guide RNA (gRNA). The base sequence of the gRNA determines the DNA-binding specificity of the fusion protein. A range of chromatin modifiers have already been engineered in this way, representing the first components of an epigenetic ‘toolbox’ (Table 1). These constructs can now be used as tools to address some fundamental questions concerning the function of individual chromatin marks.

**Table 1:** Epigenetic toolbox: a list of targetable chromatin-modifier constructs reported to date

Construct	Enzymatic function	Target region	Effect on gene expression	Reference
dCas9–p300	Histone acetyltransferase, transcriptional co-activator	Promoters and enhancers	Between approximately 50-fold (*OCT4*, *MYOD*) and 10,000-fold (*IL1RN*) upregulation when targeted to promoters (qPCR); up to 300-fold for enhancers (qPCR); lower levels of activation by RNA-seq	[8]
TALE–TET1	Methylcytosine dioxygenase (DNA demethylation)	Promoter	Between approximately 50-fold (*HBB*) and more than 100–1,000-fold (*RHOXF2*) activation over “off-target” controls (qPCR)	[7]
ZF–DNMT3a	DNA methyltransferase	Promoter	Between approximately 0.5-fold and 0.2-fold change (qPCR, western blot)	[5, 6]
ZF–G9a	H3K9 methyltransferase	Promoter	Up to approximately 0.5-fold change (qPCR)	[4]
TALE–LSD1	H3K4 and H3K9 demethylase	Several candidate enhancers	Up to approximately 0.4-fold change (RNA-seq)	[2]
dCas9–LSD1	H3K4 and H3K9 demethylase	Several candidate enhancers	Up to approximately 0.02-fold change (qPCR)	[3]
TALE–KYP, TALE–TgSET8, TALE–NUE	Histone methyltransferases	Promoter	Up to approximately 0.3-fold change (qPCR)	[10]
TALE–HDAC8, TALE–RPD3, TALE–Sir2a, TALE–Sin3a	Histone deacetylase	Promoter	Up to approximately 0.3-fold change (qPCR)	[10]
ZF + 223 different yeast chromatin modifiers	Various	Yeast pCYC1	Approximately 100-fold change (activators); approximately 0.06-fold change (repressors)	[11]

*qPCR* quantitative PCR, *TALE* TAL protein, *ZF* synthetic zinc finger

## First insights

It has long been unclear which of the large number of chromatin marks that have been catalogued over the years possess real gene-regulatory capabilities. Evidence was based mainly on statistical association of chromatin marks with expression levels of associated genes. However, it is worth bearing in mind that correlation does not necessarily imply causation. The observation that loss of chromatin modifiers causes strong phenotypes, which can often be interpreted as a consequence of transcriptional deregulation, provided further support. However, chromatin-modifier proteins also have non-histone targets, and epigenetic marks play roles in chromatin-based processes other than transcription. A causal role for chromatin marks in regulation of transcription has been convincingly demonstrated for some model loci, including the Hox and imprinted gene clusters (for example [1]), but it is still unclear whether these specific findings extend to the vast remainder of the eukaryotic genome.

By directing chromatin modifiers to a range of sites at different genomic loci and measuring resulting changes in transcription of associated candidate genes, a number of functional chromatin marks have now been identified. For example, demethylating H3K4me2 at putative enhancers with TALE–LSD1 or dCas9–LSD1 leads to downregulation of proximal genes [2, 3]. Likewise, addition of H3K9 or DNA methylation successfully silences a single gene promoter [4–6]. Removal of DNA methylation at several gene promoters using TALE–TET1 fusion proteins on the other hand results in significant upregulation of transcription [7], and similar results are achieved by histone acetylation at a handful of promoters and enhancers using dCas9–p300 [8]. These first reports hold great promise for a general applicability of epigenetic engineering, as they indicate that both activation and silencing can be achieved by single chromatin changes.

## Open questions

Collectively, these studies show that manipulation of single chromatin marks at relevant sites can significantly alter levels of transcription and that this effect depends on both the enzymatic activity of the chromatin modifier and its binding to the target site. However, some questions remain regarding the efficiency of chromatin modification using synthetic modifiers. While epigenetic editing can clearly influence transcription at a specific site, manipulation in close vicinity often produces no effect. This could reflect inherent differences in gene regulatory potential between different loci. Alternatively, it is possible that some sites cannot be modified efficiently for technical reasons (such as dCas9 binding efficiencies, transfection efficiencies), other layers of gene regulation (for example, H3K27 methylation preventing acetylation) or immediate reversal of the newly acquired change (for example, by endogenous chromatin modifiers). Thus, it remains to be addressed how many marks are functional and where in the genome these must be located in order to influence transcription.

In addition to a more detailed investigation into the efficiency of targetable chromatin modifiers, it will be important to consider whether the reported transcriptional changes are not just statistically significant, but also biologically meaningful. To some extent this is a semantic issue: while the term ‘gene activation’ suggests that gene functions have been put into effect, it can also be used to describe a minor increase in RNA levels relative to background. It would be important to compare engineered gene expression with physiological levels of expression where this is possible. In addition, analyses of protein levels [5, 6] can ensure that engineered modifications are not affecting RNA processing or stability, and that post-transcriptional processes do not compensate for transcript levels. Ultimately, it will be vital to establish the effect of epigenome engineering on cellular phenotype. Transcriptional downregulation through histone demethylation has already been reported to lead to loss of pluripotency [3]. It would be of great interest to see whether transcriptional activation of reprogramming factors, such as *OCT4* [8], through epigenome engineering can also induce cellular identity changes, or whether additional layers of gene regulation have to be manipulated before functional effects can be achieved. Future work should also elucidate the mechanistic details of how chromatin modifications exert their influence on transcription. Once the modification is added or removed from a site of interest, are transcription factors or other modifiers recruited and which are these? Which factors are required for epigenetic engineering to proceed efficiently and which are lacking if it does not?

## Looking ahead

Effectively, we now have an emerging epigenetic ‘toolbox’ for epigenome engineering, ushering in a new era of functional epigenomics. Based on the astonishing pace with which this field is moving, more chromatin modifiers will without doubt be added to the growing list of available tools. We anticipate that it will soon be possible to dissect the effect of combinations of altered chromatin marks using orthologous targeting platforms.

The ease of targeting chromatin modifiers through an RNA-based rather than protein-based DNA-binding mechanism will further enable the unbiased discovery of functional epigenetic marks using screening approaches. Screens will constitute a significant step forward from the analysis of the effect of a single mark on a single locus enabling the interrogation of many potentially functional marks in a single experiment. Libraries of guide RNAs can be constructed that are either specific to a limited range of loci of interest or potentially unbiased and genome-wide. Loss-of-function genetic screens and gain-of-function transcriptional activator screens have already been implemented [9], but no screening method has yet been reported using chromatin modifiers. Since epigenetic screens are geared towards phenotypic read-outs, they will be instrumental in addressing the open questions formulated above. Only sites where chromatin modifications have significant impact, not only on transcription, but also on phenotype, will be reported as hits.

Eventually, some of the findings may be expected to be translated into therapeutic use by adopting epigenetic engineering technologies for in vivo situations. There are already first indications that this may be feasible based on targeted epigenetic engineering of candidate promoters in the brain of living mice, positively affecting addiction-related and depression-related behavior [4]. However, to ensure that those anticipated ‘brave new epigenomes’ are not confused with the fictional ‘brave new world’, we should today, with scientific rigor and informed regulation, start discussing any ensuing ethical, legal and social implications of somatic and germline epigenetic engineering.

## Abbreviations

dCas9: Catalytically inactive version of the Cas9 protein
gRNA: Guide RNA
TALE: TAL protein
ZF: synthetic zinc finger
