# Erratum to: Brave new epigenomes: the dawn of epigenetic engineering

**DOI:** 10.1186/s13073-015-0194-7

**Published:** 2015-08-05

**Authors:** Anna Köferle, Stefan H Stricker, Stephan Beck

**Affiliations:** Medical Genomics, UCL Cancer Institute, University College London, 72 Huntley Street, London, WC1E 6BT UK; Functional Epigenetics, Institute of Stem Cell Research, Helmholtz Zentrum, German Research Center for Environmental Health, Ingolstädter Landstrasse 1, Neuherberg, 85764 Germany; Biomedical Center, Ludwig-Maximilian-Universität, Grosshaderner Strasse 9, Planegg-Martinsried, 82152 Germany

## Erratum

It has come to our attention that during the production of this article [1], the name of the first author, Anna Köferle, was incorrectly spelt as Anna Köeferle. This has been corrected in the online version of the article.
